# Synthesis of Conductive Carbon Aerogels Decorated with β-Tricalcium Phosphate Nanocrystallites

**DOI:** 10.1038/s41598-020-62822-1

**Published:** 2020-04-01

**Authors:** Atakan Tevlek, Abdulraheem M. N. Atya, Muhannad Almemar, Memed Duman, Dincer Gokcen, Alexey Y. Ganin, Humphrey H. P. Yiu, Halil M. Aydin

**Affiliations:** 1 Institute of Science, Bioengineering Division, Hacettepe University, Ankara, Turkey; 2 Institue of Science, Nanotechnology and Nanomedicine Division, Hacettepe University, Ankara, Turkey; 3 Department of Electrical and Electronics Engineering, Faculty of Engineering, Hacettepe University, Ankara, Turkey; 4 School of Chemistry, WestCHEM, University of Glasgow, Glasgow, UK; 5 Chemical Engineering, School of Engineering and Physical Sciences, Heriot-Watt University, Edinburgh, UK; 6 Centre for Bioengineering, Hacettepe University, Ankara, Turkey

**Keywords:** Biomaterials, Composites

## Abstract

There has been substantial interest in research aimed at conductive carbon-based supports since the discovery that the electrical stimulus can have dramatic effect on cell behavior. Among these carbon-aerogels decorated with biocompatible polymers were suggested as future materials for tissue engineering. However, high reaction temperatures required for the synthesis of the aerogels tend to impair the stability of the polymeric networks. Herein, we report a synthetic route towards carbon-aerogel scaffolds decorated with biocompatible ceramic nanoparticles of tricalcium phosphate. The composites can be prepared at temperature as high as 1100 °C without significant effect on the morphology of the composite which is comparable with the original aerogel framework. Although the conductivity of the composites tends to decrease with the increasing ceramic content the measured conductivity values are similar to those previously reported on polymer-functionalized carbon-aerogels. The cell culture study revealed that the developed constructs support cell proliferation and provide good cell attachment suggesting them as potentially good candidates for tissue-engineering applications.

## Introduction

The phenomenon that the electrical current can have a detrimental effect on differentiation and proliferation capacity of the cells has triggered recent research interest towards quest for biocompatible and conductive substrates^1–4^. In particular, carbon-based composite materials have emerged as potentially promising targets for growth of electroactive tissues such as cardiac muscle, nerve and bone since the stimulation of osteogenesis due to inherent piezoelectric features of bone tissues attainable on conductive scaffolds^5–9^. Among these, blended composites between nanostructured carbon materials (carbon nanofibers/nanotubes and graphene) and biocompatible polymers (poly(vinyl alcohol), alginate, poly(lactide-*co*-glycolide and collagen) have been widely investigated as future materials for tissue engineering^10–17^. However, it is often essential to use a substantial amount of nanostructured carbons as a major component for achieving high conductivity in the composites. Apart from relatively high cost (especially for carbon nanotubes) the nanostructured carbon-based materials have been shown to be toxic to cells via ATP depletion effect^18^. Therefore, the search for new and potentially less-toxic materials (which can show high level of conductivity on the range of 20–100 S m^−1^ while retaining the structural integrity) is important. In this context, aerogels (which are light-weight conductive materials and can be prepared efficiently and at low cost) present viable alternative to nanostructured carbons as a porous and conductive scaffold for a biocompatible layers^19,20^. Various inorganic and organic aerogels have been reported up to date including poly(imide)-, poly(vinyl alcohol)- and poly(vinyl chloride)-based aerogels, chalcogenide aerogels, carbide aerogels, silicon aerogels and carbon-based aerogels^21–23^. Among those carbon-based aerogels are inherently porous, display high surface area up to 800 m^2^/g and low densities often as low as 0.30 g/cm^3^ which makes them an excellent alternative to nanostructured carbons^19–21,24,25^.

Unlike nano-structured carbons, the entangled nature of carbon-based aerogels (consisting of robust and stable interpenetrated network) prevents rapid release of nanoparticles within the cell. Furthermore, they are highly conductive^26^ and thus, are potentially promising targets for applications in electroactive tissue engineering processes^27^. The major challenge is that the synthetic process of carbon-based aerogels requires high temperature synthesis (often exceeding 500 °C). The high reaction temperature prevents the implementation of commonly used biocompatible polymers and hinders the applicability of carbon-based aerogels. On the other hand, inorganic materials, such as calcium phosphates are thermally stable and have been demonstrated before as biocompatible materials for bone tissue engineering applications both *in vitro* and *in vivo*^28,29^. Among these, β-tricalcium phosphate (β-TCP) has been demonstrated as a favorable bone volume extender in the clinical applications due to the ease in sterilization and prolonged shelf life^30–34^. Combining the excellent biocompatibility of β-TCP and the conductivity of carbon aerogels can provide a viable platform for osteoconductive cell growth at low cost.

In this work, we report the synthesis of β-tricalcium phosphate (β-TCP) carbon-aerogel composite material (Graphical Abstract). We investigated the role of the pyrolysis temperature and the level of decoration with the ceramic phase on the conductivity which showed that the values as high as 25 S m^−1^ were retained in the composite networks. Moreover, the biocompatibility of the composites was evaluated and the cell growth was retained for the entire test period of 14 days. The results suggest that the future tests may reveal the composites as promising targets for biomedical applications including tissue engineering.

## Experimental

### Synthesis of porous carbon-based aerogels and composites

#### Synthesis of carbon-based aerogel

60 mg of finely shredded paper was suspended in a 40 mL 18.2 MΩ.cm electrical resistance and free from organic matter deionized water (Millipore, USA) in a beaker for 24 hours before adding 10 mL of hydrochloric acid (10% (v/v), (Fisher Scientific, USA)) and digested for 12 hours with a mechanical stirring at 1000 rpm. The resulting product was washed 4 times with deionized water and dried at 60 °C. A pulp was then prepared by mixing precursor in 30 mL of deionized water under vigorous magnetic stirring at 1000 rpm for 16 hours until a homogeneous pulp mixture was obtained. The mixture was then frozen at −80 °C in a falcon centrifuge tube and then freeze-dried (Labconco FreeZone, USA) at 0.1 atm and −86 °C to form aerogel structure. The freeze-dried sample was placed into a ceramic boat and heated at the rate of 5 °C/min under flowing argon (2 mL/min) to 850 °C or 1100 °C (see discussion section below) for 3 hours followed by natural cooling to ambient.

#### Synthesis of carbon-based aerogel/β-TCP

The β-TCP/carbon aerogel composites were prepared as described above except that β-TCP particles (BMT Calsis Co, Turkey) were added to the pulp mixture at a different ratio. The ceramic added groups were denoted as follow: 0×, 0% (w/w, ceramic/carbon); 1/2×, 16% (w/w, ceramic/carbon); 1×, 33% (w/w, ceramic/carbon); and 2×, 66% (w/w, ceramic/carbon).

### Microstructural characterization

The morphology of the obtained material was examined using scanning electron microscope (Quanta 400F Field Emission SEM, Germany). The elemental composition was evaluated by EDX analysis. To evaluate the 3D microstructural properties of the samples, a micro-CT system (Bruker SkyScan 1172, USA) was used to reveal open porosity, pore size and pore distribution (results are given in the SI). Further microstructural features of the materials were studied with transmission electron microscopy (CTEM FEI, Tecnai G^2^). For this purpose, a small amount of material dispersed in ethanol and deposited on a carbon coated copper grid.

Nano-scaled surface topography of microfibers was studied using AFM imaging with a Dimension Icon microscope (Bruker Co., CA, USA) in PeakForce Tapping mode in air. High quality etched silicon probes (RTESPW-150) for soft Tapping Mode with a nominal spring constant of 5 N/m and a resonant frequency of 150 kHz were used for AFM imaging. All images were taken by closed loop small-scan size scanner (10 × 10 μm). Integral and proportional gains were adjusted to optimize the sensitivity of the feedback loop. The sample used for AFM imaging was prepared by placing fibers onto a freshly cleaved mica surface. A FT-IR spectrometer (Thermo Nicolet IS50, USA) was used to determine the chemical compositions of the samples and the spectra were recorded in the range of 600–4000 cm^−1^ with automatic signal gain with 16 scans.

### Structural characterization

#### X-Ray diffraction

The X-ray diffraction (XRD) studies were carried out using an Ultima-IV diffractometer (Rigaku, Japan) in Bregg-Brentano configuration with Cu K_α_ radiation (40 keV accelerating voltage and a current of 30 mA) with a step size of 0.02° 1/min.

#### Raman spectroscopy

Raman spectra were recorded on an inVia-Reflex confocal Raman spectrometer (Renishaw, China) fitted with a 532 nm laser for excitation.

### Electrical characterization

Electrical conductivity measurements were conducted on the materials prepared with and without ceramic addition at 850 and 1100 °C. Since the effect of wire and contact resistances needs to be eliminated for high accuracy, particularly for the conductive materials, resistivity measurements were performed using 4-probe (wire) technique (Keithley 2450 Sourcemeter, USA). This technique is based on the concept of measuring voltage across the contacts of the sample using two wires while current passes through two other wires. For a setup given in SI-1 (Supporting Information), resistivity (*σ*) can be measured using Eq. 1

1 $$\rho =\frac{V}{I}\frac{A}{d}$$

where *V* is the voltage measured across the contacts with the distance of *d*, *I* is the current flowing over the sample, and the area of the sample cross section is given as *A*. The resistivity measurements were carried out after the samples in equal weights were fitted to equal dimensions. The electrical conductivity (*σ*) is expressed via Eq. 2.

2 $$\sigma =\frac{1}{\rho }$$

### Cytotoxicity test on *in-vitro* cell culture

In order to reveal the potential of the obtained materials for tissue engineering applications, the cytotoxicity and the cell attachment studies were conducted. The cytotoxicity test was carried out according to the instructions of the International Organization for Standardization (ISO) 10993–5: Biological evaluation of medical devices - Tests for *in vitro* cytotoxicity. For this purpose, the materials were prepared and sterilized by using 70% (v/v) ethanol solution. Sterile samples were immersed in DMEM High Glucose culture medium (Capricorn, Germany) in a concentration of 0.2 g/mL and were incubated at 37 °C in a CO_2_ incubator (Memmert, Germany) for 72 hours. At the end of the incubation period, the extraction medium was collected and filtered by using 0.22 µm filter (Millipore, USA). The extraction medium was enriched with 10% fetal bovine serum (FBS) (Capricorn, Germany), %1 antibiotic-antimycotic (ThermoFisher Scientific, USA) and %1 L-glutamine (Capricorn, Germany). In parallel to the preparation of extraction medium, P9 L929 mouse fibroblast cells (5 × 10^3^ cells/well) were inoculated to a 96-well plates containing DMEM supplemented with 10% fetal bovine serum (FBS), %1 penicillin/streptomycin (P/S) and %1 L-glutamine. Then the plates were placed in the CO_2_ incubator (37 °C in 5% CO_2_) and cultured for 24 h. The culture medium was then removed from the wells and the dilutions of extraction medium with predetermined concentrations of 2 mg/mL, 1 mg/mL and 0.5 mg/mL were applied to the cells. The culture media in the control groups were replenished with the same amount of fresh medium and the plates were cultured for another 24 hours. At the end of the culture period, the culture medium was removed and 150 µL plain DMEM High Glucose containing 15 µL MTT reagent (Sigma Aldrich, USA) was added to each well. Upon the incubation for an additional 3 h, the test solutions were discarded from the wells and 200 µL of the DMSO (Sigma Aldrich, USA) was added. Following, the plates were immediately read by using a microplate spectrophotometer (Epoch-BioTek, USA) at 570 nm wavelengths. Data were analysed with Student t test with p < 0.05 indicating statistical significance.

For the cell proliferation analysis and cell attachment study, the materials were prepared in disk shape (6 mm of diameter and 2 mm of thickness). Both studies were conducted with the 1XTCP samples pyrolyzed at 850 °C. Within this scope, 1.5 × 10^5^ MC3T3-E1 mouse pre-osteoblast cells (P19, ATCC, CRL-2593) were seeded in a 20 µL serum enriched culture medium to the materials in a 24 well plate. The cells were expanded in Alpha Minimum Essential Medium (MEM-α) supplemented with 10% FBS (Capricorn, Germany), 2 mM L-glutamine (Capricorn, Germany) and 1% antibiotic-antimycotic (ThermoFisher Scientific, USA). The cell seeded materials cultured for 14 days by using the above-mentioned culture medium. On the 1^st^, 4^th^, 7^th^ and 14^th^ day of the culture period cell proliferations were examined by using Alamar Blue solution (ThermoFisher Scientific, USA). Briefly the materials were incubated with the culture medium supplemented with 10% (v/v) Alamar Blue solution for 4 hours. At the end of the incubation period, 200 µL of the test solution from the wells were taken to a 96-well plate (n = 3 and 5 parallel) and was read at 570/600 nm by using a microplate spectrophotometer (Epoch-Biotek, USA). The obtained data were analysed with One-way Anova with p < 0.05 indicating statistical significance. For the cell attachment study, the samples were collected and fixed with 2.5% (v/v) Glutaraldehyde solution (Sigma Aldrich, Germany) for 30 min at room temperature on the 1^st^ and the 7^th^ day of the culture period. Subsequently, the fixed materials were subjected to 250 µL Hexamethyldisilazane (Sigma Aldrich, Germany) in order to make the samples completely dry. Lastly, the electron microscope images were obtained by using a SEM (Supra50VP, Germany).

## Results and Discussion

**Figure 1:**
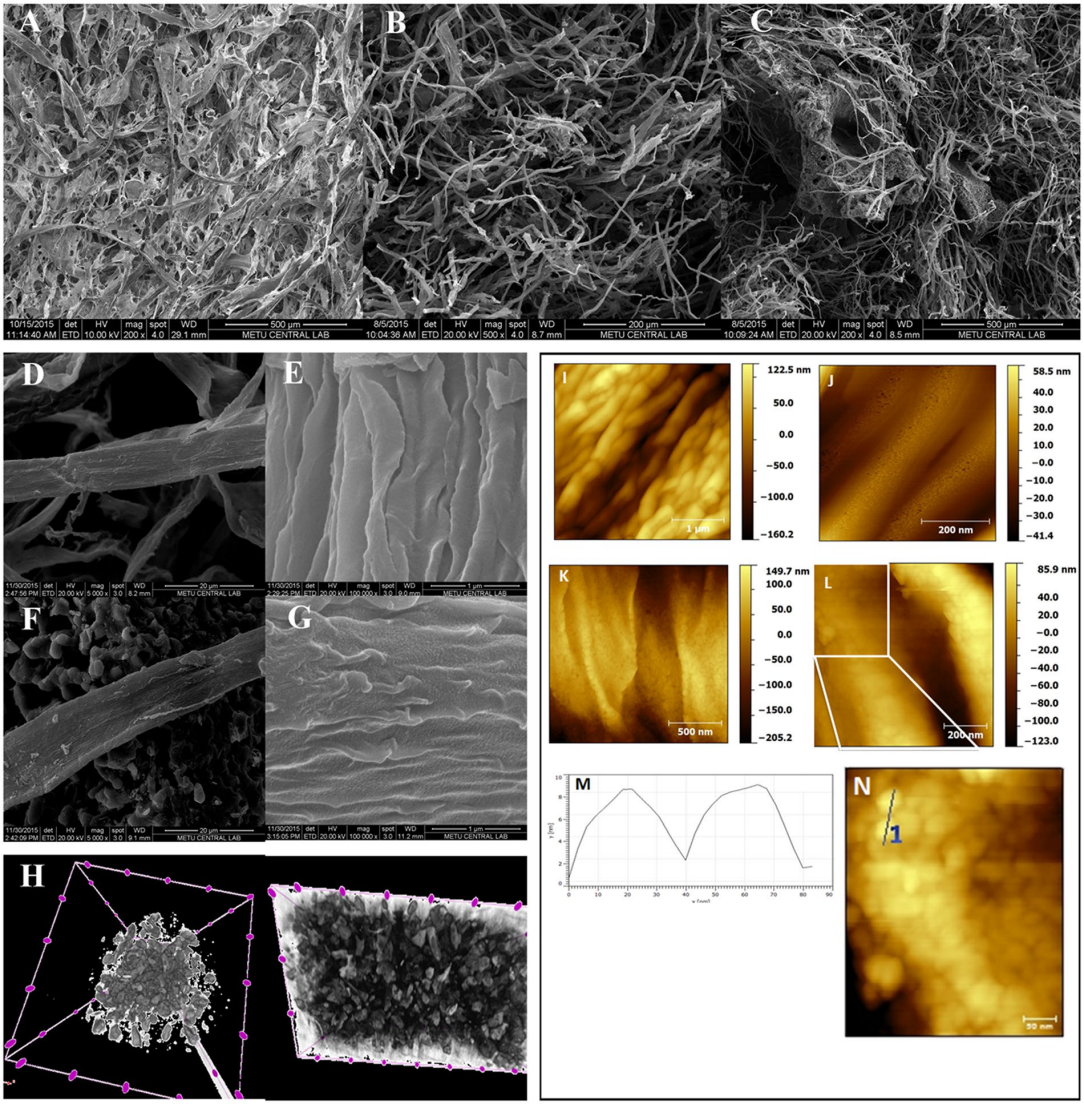
Scanning electron microscopy images of (**A**) freeze-dried precursor, (**B**,**D**,**E**) pristine carbon-based aerogel (0×), (**C**,**F**,**G**) β-TCP/Carbon aerogel (1×); (**H**) Top and side view micro-CT images of the β-TCP/Carbon aerogel (1×); (**I**,**J**) AFM images of pristine (0×) and (**K**,**L**,**N**) β-TCP decorated carbon aerogel (1×); and (**M**) line profile of the ceramic nanocrystallites.

Figure 1A shows the low magnification scanning electron microscopy (SEM) image of as made carbon-aerogel precursor after freeze-drying which consisted of belt-like fibers. The entangled network is preserved after pyrolysis procedure at 850 °C (Fig. 1B). The higher magnification images (Fig. 1D,E) confirm that the carbon aerogel sample consists of fibers with a diameter of ca. 8–10 µm, suggesting a porous material was prepared. Figure 1C,F,G show the low- and high-resolution images of the carbon aerogels functionalized with β-TCP (5000× and 100000× magnification). There is a good structural similarity between unfunctionalized and functionalized carbon aerogels, *e.g*. the addition of the biocompatible component did not change the fibrous nature of the product.

Figure 1H shows macroscopic distribution of the ceramic particles after pyrolysis. Micro-CT data of a representative β-TCP incorporated carbon aerogel (Sample 850 °C, 1XTCP) are given in SI-2 (Supporting Information). More detailed analysis on the surface of 0X and 1XTCP samples using AFM imaging showed that β-TCP loading increased the surface roughness on 1XTCP sample (Fig. 1K,L) compared to 0X sample (Fig. 1I,J). Figure 1N shows the zoom-in region confirming that β-TCP nanocrystallites are distributed homogenously on the surface of 1XTCP sample. These nanocrystallites were observed to have a disc shape with a diameter between 40 to 60 nm (Fig. 1L,N). Line profile analyses from the AFM image (Fig. 1M) revealed that the average height of these nanocrystallites was around 6 ± 1 nm (n = 50). SI-3 (Supporting Information) also gives similar information, calculated using ImageJ software (1.52a, USA). Representative TEM images can also be found in SI-4 (Supporting Information).

**Figure 2:**
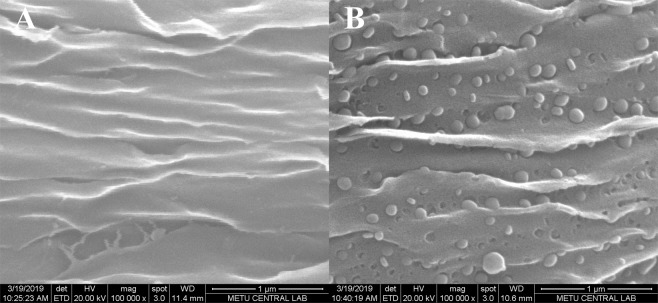
Scanning electron micrographs of microbelt surface of the 1XTCP samples pyrolyzed at 850 °C (**A**) and 1100 °C (**B**).

Figure 2A shows a magnified region of the composite sample prepared at 850 °C. The area appears to be smooth compared to that observed for the same sample pyrolyzed at 1100 °C (Fig. 2B), which may indicate that the surface of carbon aerogel template is covered homogeneously by the β-TCP layer at lower pyrolysis temperature utilized.

**Figure 3:**
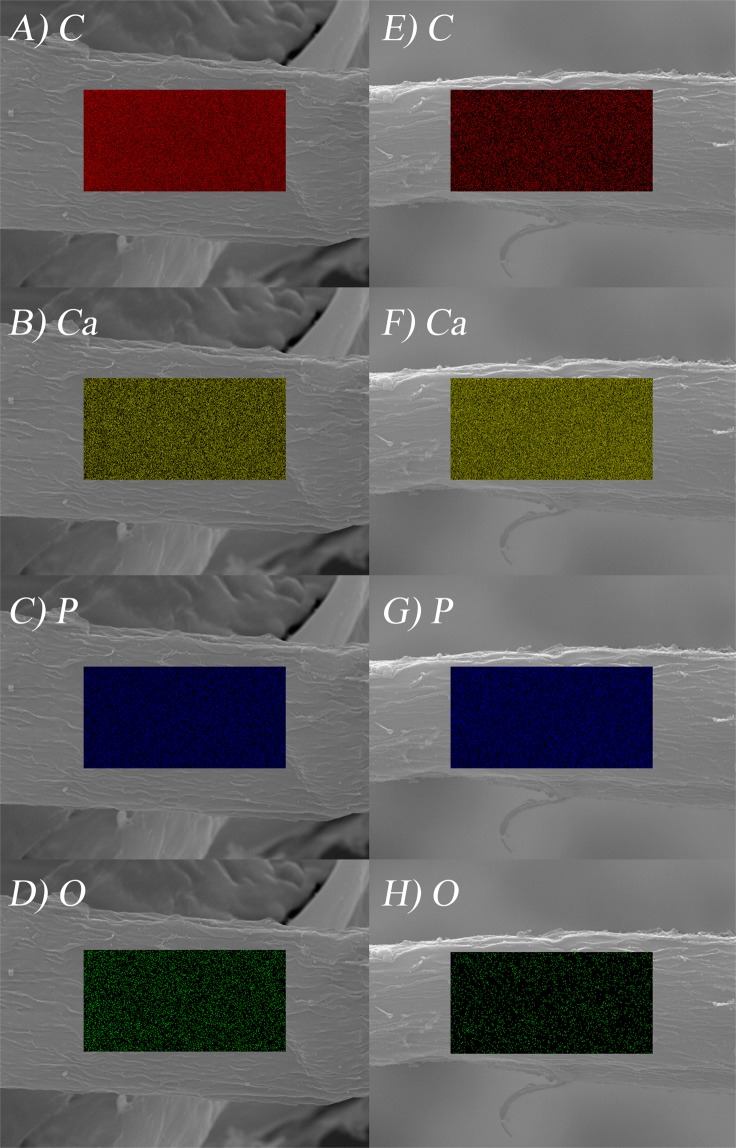
EDX mapping for C, Ca, P and O on the microbelt surfaces on the samples pyrolyzed at 850 °C (**A**–**D**) and 1100 °C (**E**–**H**).

Energy Dispersive X-Ray Spectroscopy (EDX) mapping was used to probe in to evaluate the distribution of Ca and P to confirm the presence of β-TCP layer on the surface of carbon aerogels. The maps for Ca, O, and P reveal that these elements are homogeneously distributed on the carbon (Fig. 3A,E) fiber surface (Fig. 3B,D,H,F,C,G, respectively). In addition, the calcium to phosphorus (Ca:P=1.5) molar ratios determined from the elemental analysis (SI-5, Supporting Information) agrees well with the chemical formula Ca_3_(PO_4_)_2_. While EDX can provide good evidence for elemental distribution, it provides no information about crystal structure of the phosphate. Therefore, we collected a powder X-ray diffraction pattern on a composite product which is in a perfect agreement with the simulated pattern of β-TCP (SI-6 & SI-7, Supporting Information).

**Figure 4:**
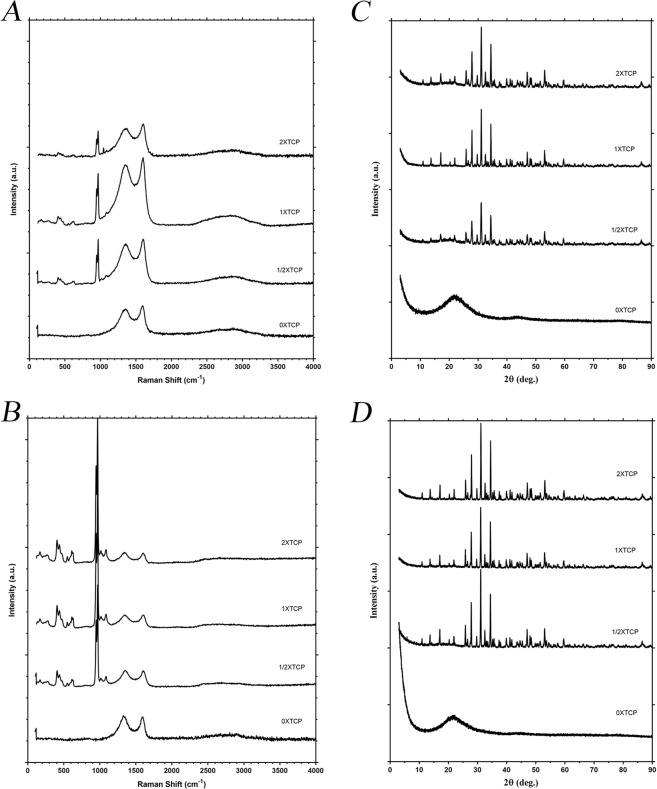
Raman spectra ((**A**) 850 °C, (**B**) 1100 °C) and XRD patterns ((**C**) 850 °C, (**D**) 1100 °C) of the β-TCP/carbon aerogel materials with various β-TCP amounts.

Raman spectroscopy spectra were recorded to qualitatively compare the level of graphitization (an important factor to consider for improved conductivity) between the pristine carbon aerogel and composite materials (Fig. 4A,B). The Raman spectra display two pronounced peaks at *ca*. 1587 cm^−1^ (G-band) and *ca*. 1340 cm^−1^ (D-band) suggesting C-C bonds corresponding to the conjugated bond (similar to the one observed in graphite) and the bonds associated with defects or disorder within the graphitic-like structures, respectively. The intensity ratio between the D and G peaks (*I*_*D*_*/I*_*G*_), which is linked to the degree of disorder^35^, were found to be 0.88 and 0.91 for the carbon aerogel and composite respectively which indicates a good similarity between two products. In addition, a broad peak associated with sp^2^ bonding and ascribed to 2D-planes is recognizable 2500–2800 cm^−1^ ^36–38^. Overall, visually there is a little difference between the Raman spectra of the carbon aerogel (Fig. 4A) and the composite (Fig. 4B). The peaks in the composite at 432 cm^−1^, 610 cm^−1^ and 966 cm^−1^ are corresponding to spectral line expected for β-TCP and thus, further confirming that the surface of the carbon aerogel is functionalized by the ceramic material. The FTIR spectra of the aerogels also confirmed prepared structures (SI-8, Supporting Information).

X-ray diffraction (XRD) studies on the prepared samples were also carried out to evaluate the structure of the composite materials and to determine the crystallite sizes of the β-TCP formed on these fibers. For both 0X samples, pyrolyzed at 850 and 1100 °C, the XRD patterns showed a broad band at 2θ = 22.5° which was related to its amorphous graphitic nature (Fig. 4C,D)^39,40^. These signals were not observed in other ceramic loaded samples as they were shielded by those of the highly crystalline β-TCP in the same 2θ range^41^. The other peaks were found to be related to those for β-TCP according to XRD database. Scherrer analysis was performed to determine the mean size of the nanocrystallites formed on the carbon fibers (see SI-9, Supporting Information). The mean domain size was found similar for 1XTCP subgroups in 850 and 1100 °C samples (58 nm and 59 nm).

**Figure 5:**
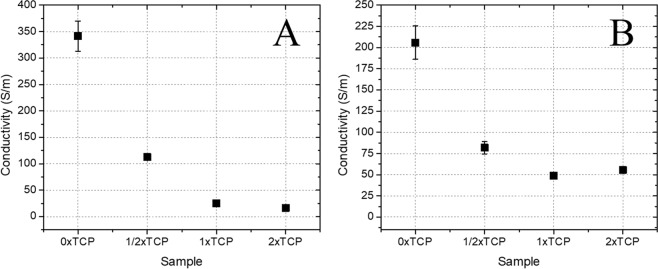
Conductivity values for composite samples depending on the amount of β-TCP addition. Samples prepared at 850 °C (**A**) and 1100 °C (**B**).

Finally, the conductivity values for carbon aerogels and composite materials were also examined using a 4-probe method (Fig. 5A,B). Compared with the pristine carbon aerogel material (which demonstrated an impressive level of conductivity of 341 ± 28 S m^−1^) there is more than an order of magnitude reduction in the conductivity value for the composite material. The observed value of 25.13 ± 0.97 S m^−1^ is however, comparable to the literature results reported for nanocarbon materials functionalized with biocompatible polymers. For example, Guex *et al*. have recently reported a conductivity value of 0.140 S m^−1^ for a poly(3,4-ethylenedioxythiophene):poly(styrene sulfonate) PEDOT:PSS based porous scaffold^5^. More recently, Chen *et al*.^1^, a carbon aerogel was prepared via a similar route with a conductivity value of 32.6 S m^−1^. The authors used dopamine coating which required an additional post-functionalization step after pyrolysis. The approach used in this work allows for fabrication of the composite directly on the carbon aerogel without additional post-modification step. In addition, the conductivity can be tuned by varying the amounts of carbon aerogel: β-TCP ratios.

**Figure 6:**
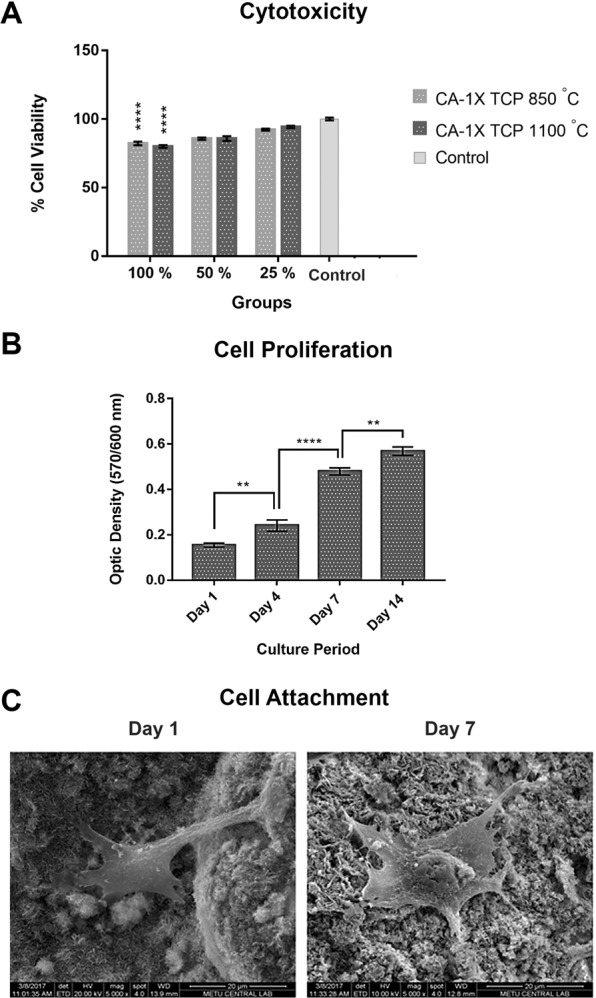
Cell viability according to the *in vitro* cytotoxicity test (**A**), MC3T3 mouse pre-osteoblast cell proliferation on 1XTCP 850 °C (**B**) and MC3T3-E1 cell behavior on 1XTCP 850 °C (**C**).

Dose dependent *in vitro* cytotoxicity analysis was performed using a composite material in order to determine whether the developed materials were cytotoxic. Assuming that the viability of the control cells was 100%, the cell viability values in the other groups were expressed in percent of the control group and are presented in Fig. 6A. The viability values of the cells treated with the 100% extraction medium in the group of 1XTCP 850 °C were recorded as 82.22 ± 1.33% respectively (****p < 0.0001). When the concentration of the extraction medium was 50% diluted, the viability values were calculated as 85.65 ± 0.85%. Finally, a minimum of 92.30 ± 0.7% cell viability was observed in the groups in which the extraction medium was diluted 75%. The materials are considered cytotoxic when the cell viability was calculated below 70% in light of the cytotoxicity test results according to the ISO-10993 standard^42^. In this context, when the data obtained from all the groups in this study were evaluated, the cell viability which was recorded as minimum 80 ± 0.25% indicates that the materials produced were not exhibited any cytotoxic effects.

The cell proliferation study was performed in addition to the cytotoxicity analysis and the results were given in Fig. 6B. According to the obtained graph, it was shown that the cells exhibited proliferation regularly on the material surface from the first day to the 14^th^ day of the culture. An approximately 1.5-fold increase was observed in the cell viability from day 1 to day 4 of the culture period (**p < 0.01), while 2-fold increase was observed from day 4 to day 7 (****p < 0.0001). Lastly, a subtle increase was also noted in the cell viability after the 7^th^ day of the culture until day 14 (**p < 0.01). These results suggested that the cells were adapted to the microenvironment easily and were exhibited a tendency to grow. On the other hand, cellular behavior on these materials were observed on 1^st^ and 7^th^ day of the culture period besides cell proliferation and is given in Fig. 6C. It was observed that the cells were exhibited their original morphology on the materials on both 1^st^ and 7^th^ day of the culture period. The positive observation from the cellular behavior here was suggested to be due to the presence of the β-TCP found in the carbon fibers. Previous literature on the potential of the β-TCP for osteoblasts culture and bone remodeling through its chemical and physical properties support this suggestion^43,44^. Therefore, the developed ceramic nanocrystallite decorated surface improves cellular behavior. Even though there have been reports of toxicity for nanoparticulate β-TCP, the osteoinduction capabilities thereof are also very well documented in providing calcium source in the nucleation processes throughout bone healing^45,46^.

## Conclusions

A conductive, porous, non-toxic, and biocompatible scaffolds from carbon aerogels with ceramic nanocrystallite decorated fibers were produced. These scaffolds are conductive and the conductivity value can be tailored by playing the process parameters. In contrast to the current trends where carbon materials are incorporated into polymer matrices to produce conductive scaffolds for tissue engineering applications, here we demonstrate a facile route where tuning the conductivity of the final constructs is achieved by introducing a dielectric and compatible material. The materials developed from this study have shown good biocompatibility and supported cell growth for at least 14 days. These may be potential candidates for compatible conductive scaffolds to be used in tissue engineering and among other biomedical applications.

## Supplementary information

Supplementary Information.
