# Supplementary material for: Synthesis of Conductive Carbon Aerogels Decorated with β-Tricalcium Phosphate Nanocrystallites
Source: Sci Rep. 2020 Apr 1;10:5758. doi: 10.1038/s41598-020-62822-1 (PMC7113289; doi:10.1038/s41598-020-62822-1)
Supplement: Supplementary file 1 — Supplementary Information. [file 41598_2020_62822_MOESM1_ESM.pdf]

Supporting Information for

Synthesis of Conductive Carbon Aerogels Decorated with β-Tricalcium Phosphate Nanocrystallites

Atakan Tevlek[1], Abdulraheem M. N. Atya[1], Muhannad Almemar[1],

Memed Duman[2], Dincer Gokcen[3], Alexey Y. Ganin[4],

Humphrey H. P. Yiu[5] and Halil M. Aydin[1,6]

Index	*Page*
SI-1	2
SI-2	2
SI-3	3
SI-4	3
SI-5	4
SI-6	4
SI-7	5
SI-8	5
SI-9	6

SI-1. Schematic representation of the set-up for 4-probe measurement system.

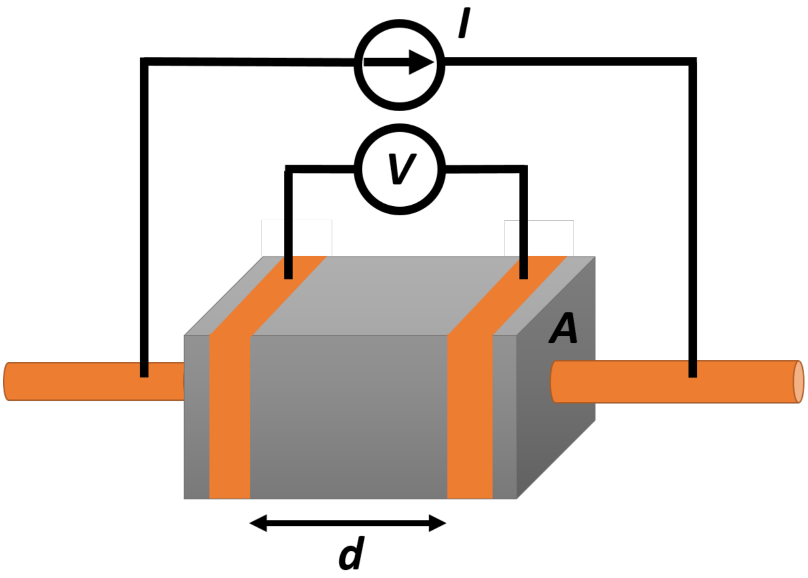

SI-2. Micro-CT data of a representative β-TCP incorporated (Sample 850 °C 1XTCP) carbon aerogel structure.

**Parameter**	**Value**
Total Porosity	90.80 %
Open Porosity	90.75 %
Surface Area	795.14 mm^2/sample
Surface/Volume Ratio	16.79 1/mm
Connection Density	1.49 1/mm^3

SI-3: SEM images shows the β-TCP nanocrystallite sizes on microbelts (Sample 1100 °C 1XTCP):

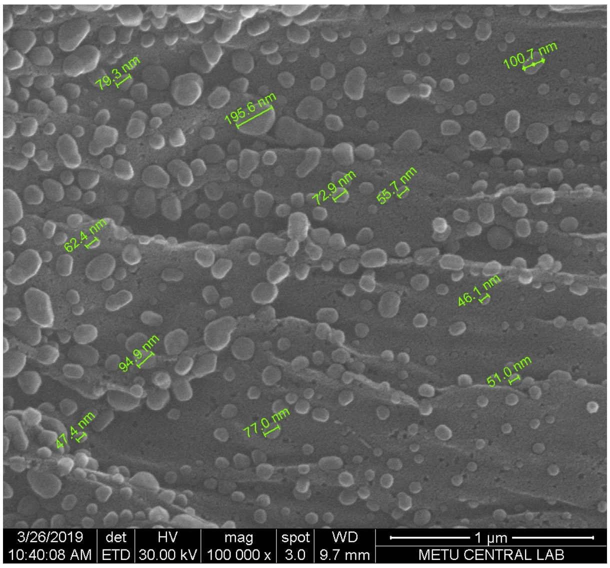

**Particle Size calculated by using Image J Software (1.52a USA)**	
Mean	74.76 nm
S.D.	30.17 nm
Minimum	30.27 nm
Maximum	199.33 nm

SI-4: TEM images of the constructs: carbon aerogels without (left) (Sample 850 °C - 0XTCP) and with (right) β-TCP (850 °C - 1XTCP):

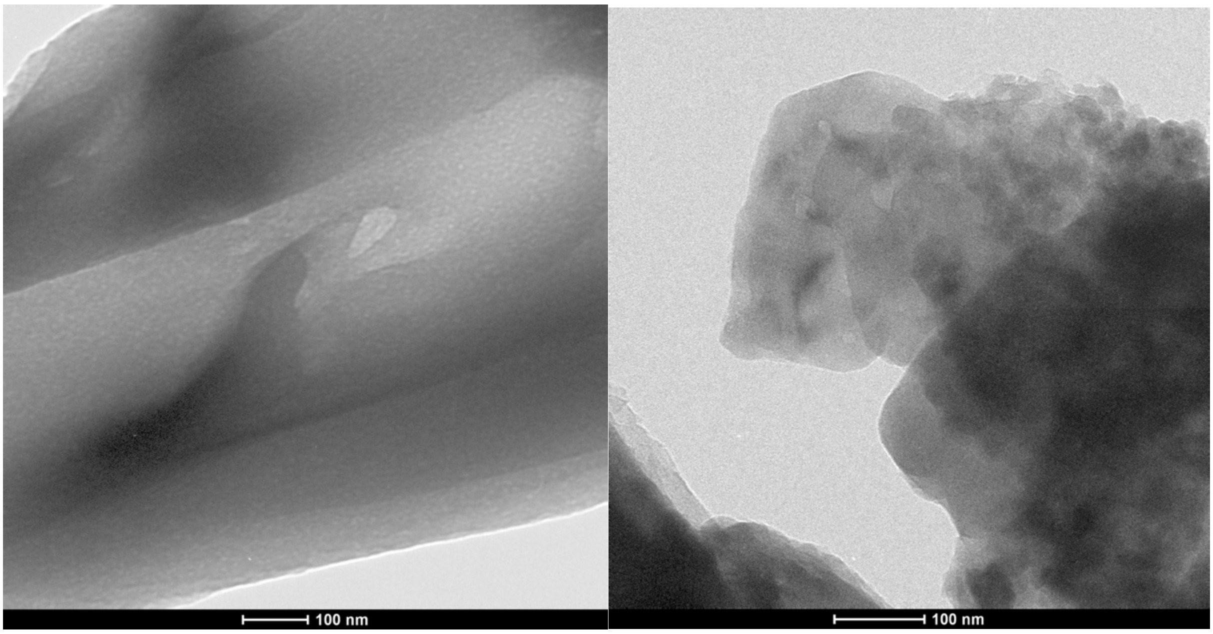

SI-5: EDX atomic percentages (Sample 1100 °C 1XTCP).

**Element**	**Atomic %**
Calcium	2.79
Phosphorus	1.85
Oxygen	6.15
Ca/P Ratio	*1.5081*

SI-6: XRD diffractogram of β-TCP particles used in the synthesis step.

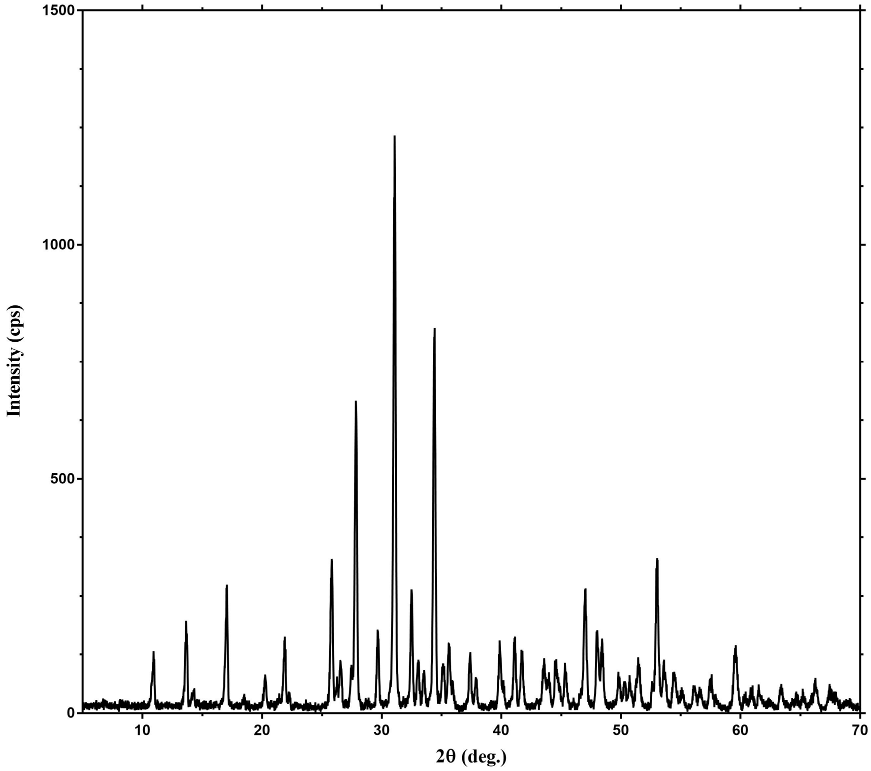

SI-7: XRD diffractogram of a reference β-TCP from the library.

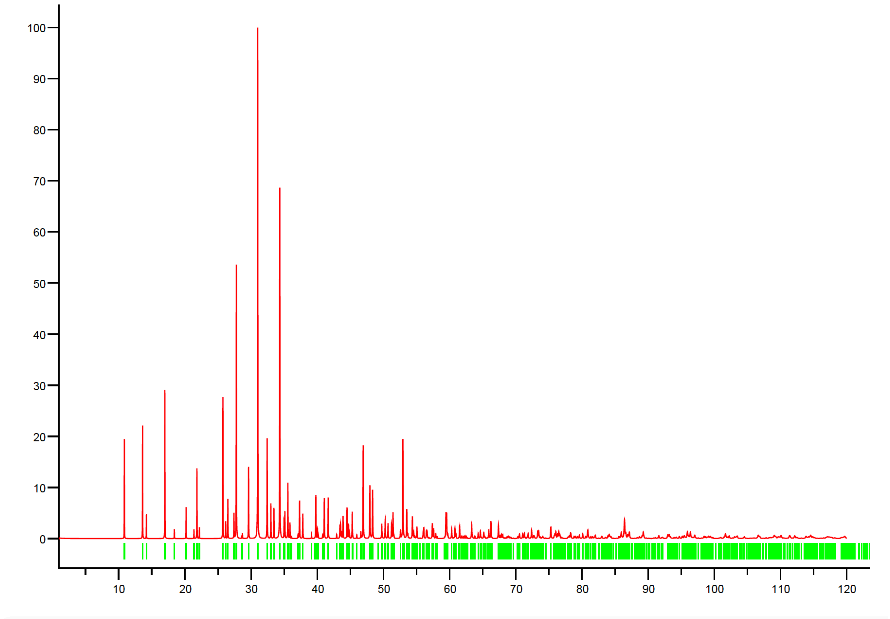

(Source: http://icsd.cds.rsc.org/search/basic.xhtml)

SI-8. FTIR spectra of pure β-TCP particles and carbon aerogels (C.A.) with (1XTCP) and without (0XTCP) ceramic phase, both pyrolyzed at 850 °C.

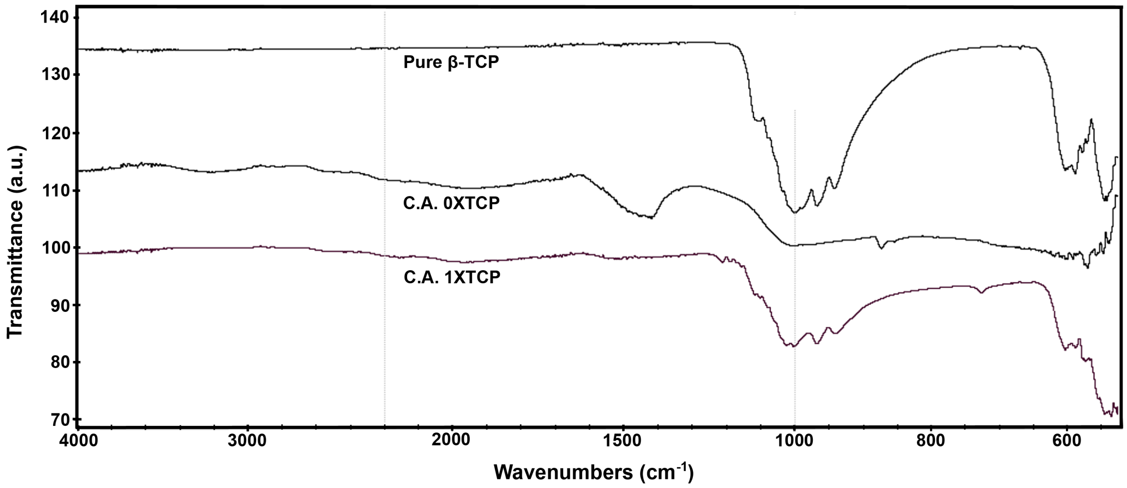

The FTIR spectra of the prepared aerogels were analyzed. The broad band at around 3200 cm^{-1} (stretching vibrations) and the band at around 1425 cm^{-1} (bending vibrations) were due to the existence of $–CH_3$, $-CH_2-$, =CH- groups in the structure of pyrolysis products without β-TCP. For the case of β-TCP containing pulp pyrolysis, the signals representing C-H bonds completely disappeared due to the existence of three σ and one π bonds between only carbon atoms. Additionally, broad and weak C=C stretching vibrations at around 1565 cm^{-1} and weak bending vibrations at around 725 cm^{-1} also observed in the structure of the pyrolyzed pulp even though it contains three-fold higher amounts of β-TCP than the paper. Therefore, the broad band around 1000 cm^{-1} and the peaks at 600 cm^{-1} and 540 cm^{-1} shows the existence of β-TCP, namely symmetric stretching mode ν_1, vibrational modes ν_4 and ν_3, respectively, in addition to the interfered IR signals of carbon material formed in the structure of the same pyrolyzed sample.

SI-9. Mean size values of the ordered domains of β-TCP ceramic nanocrystallites on the carbon aerogel fibers by XRD.

**Sample**	**Mean FWHM**	**Calculated Crystallite Size (nm)**
850 °C 1/2XTCP	0.265	35.01
850 °C 1XTCP	0.159	58.38
850 °C 2XTCP	0.219	42.40
1100 °C 1/2XTCP	0.157	59.75
1100 °C 1XTCP	0.158	58.75
1100 °C 2XTCP	0.151	61.51

*Scherer Equation **($\tau = K\lambda/\beta\cos\theta$)** was used in the calculations. Shape factor K was taken as 0.9 while **λ** was taken as 0,15406 nm. β, the broadening at the FWHM intensity (radians) and Bragg angle (θ) was given in the table. The highest and characteristic three Bragg angle values were selected. 3 Bragg angles used for each diffractogram (e.g. 2Tetha=31.11°, 28.02°, 34.5°).
